# The Impact of Blood and Urine Biomarkers on Age-Related Macular Degeneration: Insights from Mendelian Randomization and Cross-sectional Study from NHANES

**DOI:** 10.1186/s12575-024-00248-z

**Published:** 2024-06-25

**Authors:** Wang Jingzhi, Xuehao Cui

**Affiliations:** 1 Department of Radiotherapy Oncology, Affiliated Hospital of Medical School, Nanjing University, Yancheng No.1 People’s Hospital, Yancheng, China; 2 John Van Geest Centre for Brain Repair and MRC Mitochondrial Biology Unit, Department of Clinical Neurosciences, University of Cambridge, Cambridge, CB2 0PY UK; 3 Cambridge Eye Unit, Addenbrooke’s Hospital, Cambridge University Hospitals, Cambridge, UK

## Abstract

**Introduction:**

Age-related macular degeneration (AMD) is a leading cause of blindness, affecting millions worldwide. Its complex pathogenesis involves a variety of risk factors, including lipid metabolism and inflammation. This study aims to elucidate the causal relationships between biomarkers related to these processes and AMD, leveraging Mendelian randomization (MR) and cross-sectional analysis from the National Health and Nutrition Examination Survey (NHANES).

**Method:**

We conducted a two-phase study, initially using MR to explore the causality between 35 biomarkers and various AMD subtypes, followed by observational analysis with NHANES data to validate these findings.

**Results:**

MR analysis identified a protective role of TG and a risk factor role of HDL-C and CRP in AMD development. NHANES data corroborated these findings, highlighting a nuanced relationship between these biomarkers and AMD. Notably, lipid metabolism-related biomarkers showed stronger associations with early AMD, whereas CRP’s significance was pronounced in late AMD.

**Conclusion:**

This comprehensive analysis, combining MR with NHANES data, reinforces the importance of lipid metabolism and inflammation in AMD’s etiology. Future research should further investigate these biomarkers’ mechanisms and their potential as therapeutic targets for AMD prevention and treatment.

**Supplementary Information:**

The online version contains supplementary material available at 10.1186/s12575-024-00248-z.

## Introduction

Age-related macular degeneration (AMD), a leading cause of global blindness, currently affects 196 million people and is projected to impact around 288 million by 2040 [1]. This disease manifests in two forms: neovascular AMD (wet AMD, wAMD) and non-neovascular AMD (dry AMD, dAMD) [2]. Dry AMD is the more prevalent variant and may evolve into wet AMD [3]. Wet AMD, despite being less common, is responsible for approximately 80% of the severe vision impairment associated with AMD due to retinal hemorrhage and exudation [4]. A multitude of risk factors contributing to AMD’s intricate pathogenesis have been identified, including advanced age, tobacco use, elevated body mass index, hypertension, hyperlipidemia, and genetic predispositions [5]. Of these, age is considered the most significant risk factor. The elucidation of these mechanisms has facilitated the advancement of targeted therapeutic and preventative strategies for AMD [6]. The diagnosis of AMD primarily depends on fundus examinations. With technological advancements, tools such as fundus photography, optical coherence tomography (OCT), OCT angiography (OCTA), and fluorescein angiography (FFA) can be utilized to assess AMD at different stages and subtypes. However, the symptoms of early AMD, whether dry or wet, are highly inconspicuous and difficult to detect [7].

Laboratory testing, involving the analysis of blood and urine biomarkers, are frequently measured to diagnose and monitor chronic disease conditions. In recent years, researchers have identified mechanisms such as lipid metabolism, systemic inflammation, autophagy, complement regulation, and mitochondrial dysfunction as having a causal relationship with the onset of AMD, and these mechanisms can be explored through a series of biomarkers [8–12]. A previous studies show that higher levels of certain lipids like high-density lipoprotein cholesterol (HDL-C) and apolipoprotein a1 (ApoA1) increase the risk for all types of AMD, while lower levels of other lipids, notably low-density lipoprotein cholesterol (LDL-C) and ApoB, along with triglycerides (TG), are linked to a lower risk for specific AMD subtypes, a finding that holds up even after accounting for lipid interactions [13]. Another research indicates that C-reactive protein (CRP), serving as a mediator of complement activation and inflammatory signaling in AMD, plays a role in the development of AMD [14]. A study revealed an association between the concentration of plasma lipoprotein subfractions and both lipid metabolism and AMD, indicating that a variety of blood and urine biomarkers found in laboratory tests may be causally linked to AMD [15]. Identifying these biomarkers could significantly enhance the diagnosis and treatment of AMD.

Mendelian randomization (MR) is a statistical method that leverages genetic variations to investigate causal links between factors and disease risks, overcoming some traditional observational study challenges like unmeasured confounding [16, 17]. By using genetic variants as tools to isolate the influence of specific exposures free from environmental or other trait influences, MR aligns closely with the principles of randomized controlled trials in its evidentiary value [18]. MR has shown promise in elucidating connections between AMD and various factors like a serious of biomarkers. The UK Biobank (UKB) conducted lab tests on over 30 serum and urine biomarkers for a cohort of over 480,000 people, inclusive of unrelated individuals, gathering comprehensive phenotype and genotype data [19]. In this study, we employed a systematic MR to explore the causal relationships between 35 biomarkers and various subtypes and stages of AMD. Subsequently, by utilizing data from the National Health and Nutrition Examination Survey (NHANES), we investigated the association between these biomarkers and AMD, further validating their clinical significance.

## Method and Materials

### Study Design

**Fig. 1:**
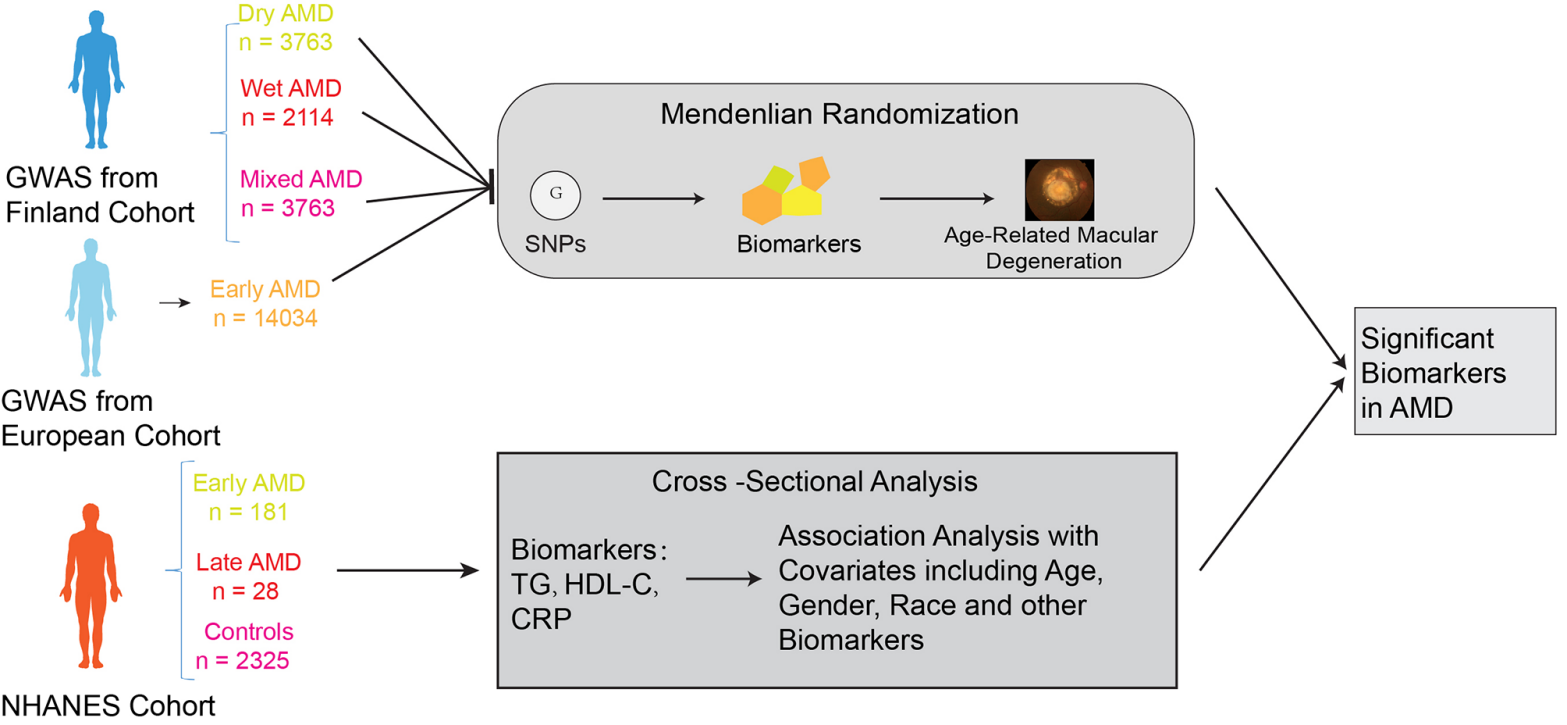
The study design and review

This study was carried out in two phases, as illustrated in Fig. 1. In the first phase, we employed MR to investigate the causal relationships between 35 biomarkers and dry, wet, early, and mixed types of AMD. To ensure the validity of the MR analysis, three criteria had to be met: (1) The genetic variants used in the analysis must be significantly associated with the exposure; (2) the genetic variants chosen as instrumental variables (IVs) for the exposure must not be correlated with confounding factors that influence both the exposure and outcome; and (3) there must be no horizontal pleiotropy, meaning the IVs can only affect AMD through the exposure mechanism [20]. In the second phase, we conducted an observational study using data from the NHANES, which serves as a foundational resource for health monitoring among the US civilian population. We verified the biomarkers identified as having a causal relationship with AMD in the MR study within the NHANES dataset’s patient cohort, confirming a significant association between these biomarkers and AMD.

### Data Source and Study Population in MR

The UKB is a substantial prospective cohort study which enlisted over 500,000 participants aged 40 to 69 years from 22 assessment centers throughout the UK between 2006 and 2010 [21]. To mitigate potential confounding factors related to ancestry, our analyses were limited to unrelated individuals of European descent [19]. We conducted a comprehensive examination and precise fine mapping of loci associated with 35 biomarkers in a cohort of 363,228 individuals. In this study, four AMD phenotypes were utilized as outcome measures. The Genome-Wide Association Study (GWAS) of early AMD was derived from a meta-analysis including individuals of European descent, incorporating data from 10 studies. A total of 14,034 early AMD cases and 91,214 controls were included in this data. The GWAS for the other three AMD phenotypes, including dry, wet, and mixed types, all originated from the the FinnGen Release 10 (R10), which is a genotype dataset from Finnish biobanks including more than 500,000 individuals. In the datasets for AMD, there are 6,651, 5,239, and 9,721 cases respectively in dry, wet and mixed AMD, while the control groups consist of 272,504, 273,920, and 381,339 individuals respectively.

### Instrument Selection of MR

We adopted a careful approach for selecting instrumental variables, initially setting a *p*-value threshold of > 5E − 08 to identify appropriate single-nucleotide variants (SNVs) for use as instruments. To ensure that only uncorrelated variants were included in MR analysis, all variants correlated with the most signifcant SNPs were excluded (clumping r^2^ cut-of = 0.001, clumping window = 10,000 kb).

### MR Analysis

For two-sample MR, the effect of biomarkers on AMD subtypes was assessed using the inverse variance weighted (IVW) method with a random-effects model in TwoSampleMR v.0.5.6 (https://mrcieu.github.io/TwoSampleMR/). Data harmonization and analyses were conducted using the TwoSampleMR package version 0.5.6. MR analysis was performed with the ‘mr()’ function, applying an FDR-P value correction by Benjamini-Hochberg (BH) method to enhance the statistical significance. Heterogeneity was assessed using the ‘mr_heterogeneity()’ for heterogeneity *P* values, with a heterogeneity *P* value (Q_pval) < 0.05 indicating substantial heterogeneity. To assess directional pleiotropy, we employed the MR-Egger intercept test. Directional pleiotropy was deemed present if the MR-Egger intercept significantly deviated from the null (*P* < 0.05). A calculated F-statistic (F > 10) was to test whether each SNP is strongly associated with exposure. We employed MR-Egger, weighted median, and weighted mode methods as supplementary analyses to assess the effect combined with the IVW method. We conducted reverse MR analysis on these biomarkers with causal associations to exclude bidirectional interactions between exposure and outcome.

### Data Sources and Study Population of NHANES Analysis

NHANES is a continuous cross-sectional series of surveys conducted on non-institutionalized U.S. civilians. Utilizing multistage probability sampling, it selects a nationally representative sample and assesses their health and nutritional status through household interviews, physical examinations, and laboratory tests. Administered by the National Center for Health Statistics of the Centers for Disease Control and Prevention (CDC), details about the sampling method and data collection can be found in a previous publication. All statistical analyses were performed considering the NHANES complex survey design, incorporating weights, stratification, and clustering to ensure that our estimates reflect the broader U.S. population. This approach enhances the generalizability of our results and strengthens the validity of our findings in the context of population-based health research. The study received approval from the Ethics Review Board of the National Center for Health Statistics, and all participants provided written informed consent [22].

To investigate the association between biomarkers and AMD subtypes, we used publicly available NHANES survey data from the 2005/2006 and 2007/2008 cycles. Initially, we identified a total of 7,081 participants aged 40 years and older. Subsequently, we excluded 1,253 participants due to a lack of necessary retinal photographs and an additional 337 participants due to missing information on the classification of AMD severity. Subsequently, based on the results of the previous MR analysis, we identified triglycerides, HDL-C, and CRP as the focus of our study to investigate their association with AMD. Next, based on the results of the previous MR analysis, we identified triglycerides, HDL-C, and CRP as the focus of our study to investigate their association with AMD. We excluded 2,957 participants who lacked information on these three biomarkers. After these exclusions, a final cohort of 2,534 participants was identified (Fig. 1). The assessment of retinal photographs, available on the NHANES website, was meticulously conducted by at least two experienced experts using a rigorous procedure for the diagnosis and classification of AMD [23]. Among them, there were a total of 209 AMD patients and 2,325 controls, with 181 participants classified as early AMD and 28 confirmed as late-stage AMD. In the NHANES surveys, participants were required to provide written informed consent before enrollment.

### Covariates in Analysis

The regression models were adjusted for covariates previously associated with biomarkers and AMD, including age, gender, race/ethnicity (categorized into five groups), education level (categorized as those who had completed less than high school, completed high school or graduate equivalency degree, and completed more than high school), body mass index (BMI), history of hypertension, and diabetes mellitus. Additionally, other potentially relevant indicators such as ApoB, HbA1C, cholesterol (CHO), LDL-C, albumin (ALB), and insulin were incorporated.

### Statistical Analyses

In our study, we utilized EmpowerStats software and logistic regression models for the analysis of clinical data. The baseline characteristics of the study population were statistically described by AMD subgroups. Continuous variables were reported as mean values with standard deviation (SD) and analyzed using weighted linear regression models. Beta values and 95% confidence intervals were determined through multivariate linear regression analysis to assess the association between biomarkers and AMD. Multivariate testing comprised three models: model 1 with no adjusted variables, model 2 adjusted for gender, age, and race, and model 3 adjusted for all covariates. Smoothed curve fits were concurrently adjusted for variables. A threshold effects analysis model was utilized to investigate the relationship and inflection point between biomarkers and AMD. The same statistical methods were applied to gender subgroups. Statistical significance was set at *P* < 0.05. We employed a weighting approach to mitigate significant volatility in the dataset.

## Result

### Causal Effect of Biomarkers with AMD in MR

**Fig. 2:**
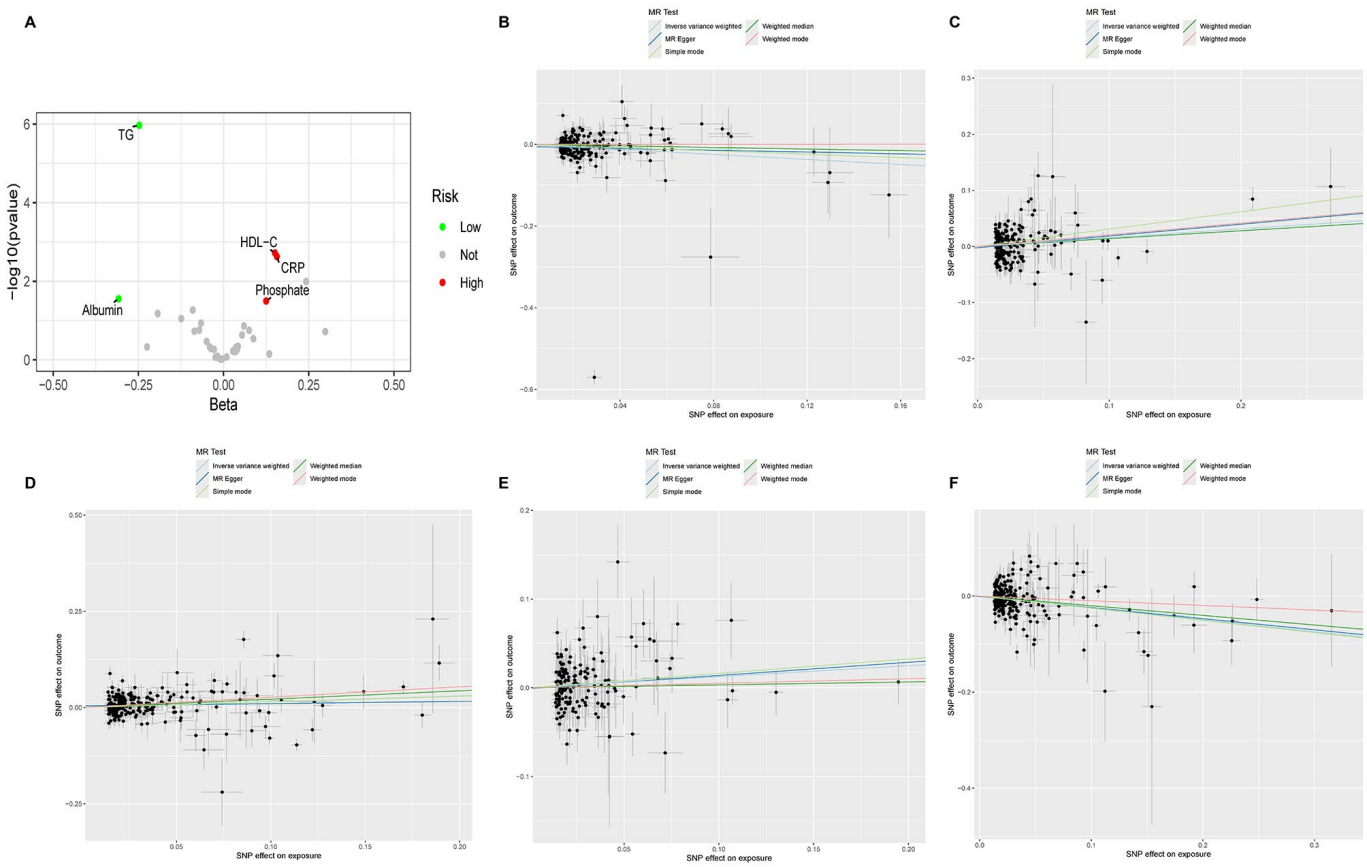
The volcano plot and scatter plot of biomarkers. **A** The volcano plot shows the most significant biomarkers with AMD. The red dots are biomarkers with positive effects with AMD and the green dots are negative. The grey dots are biomarkers without significant effect. **B** The scatter plot of ALB with AMD. **C** The scatter plot of CRP with AMD. **D** The scatter plot of HDL-C with AMD. **E** The scatter plot of Phosphate with AMD. **F** The scatter plot of TG with AMD

**Fig. 3:**
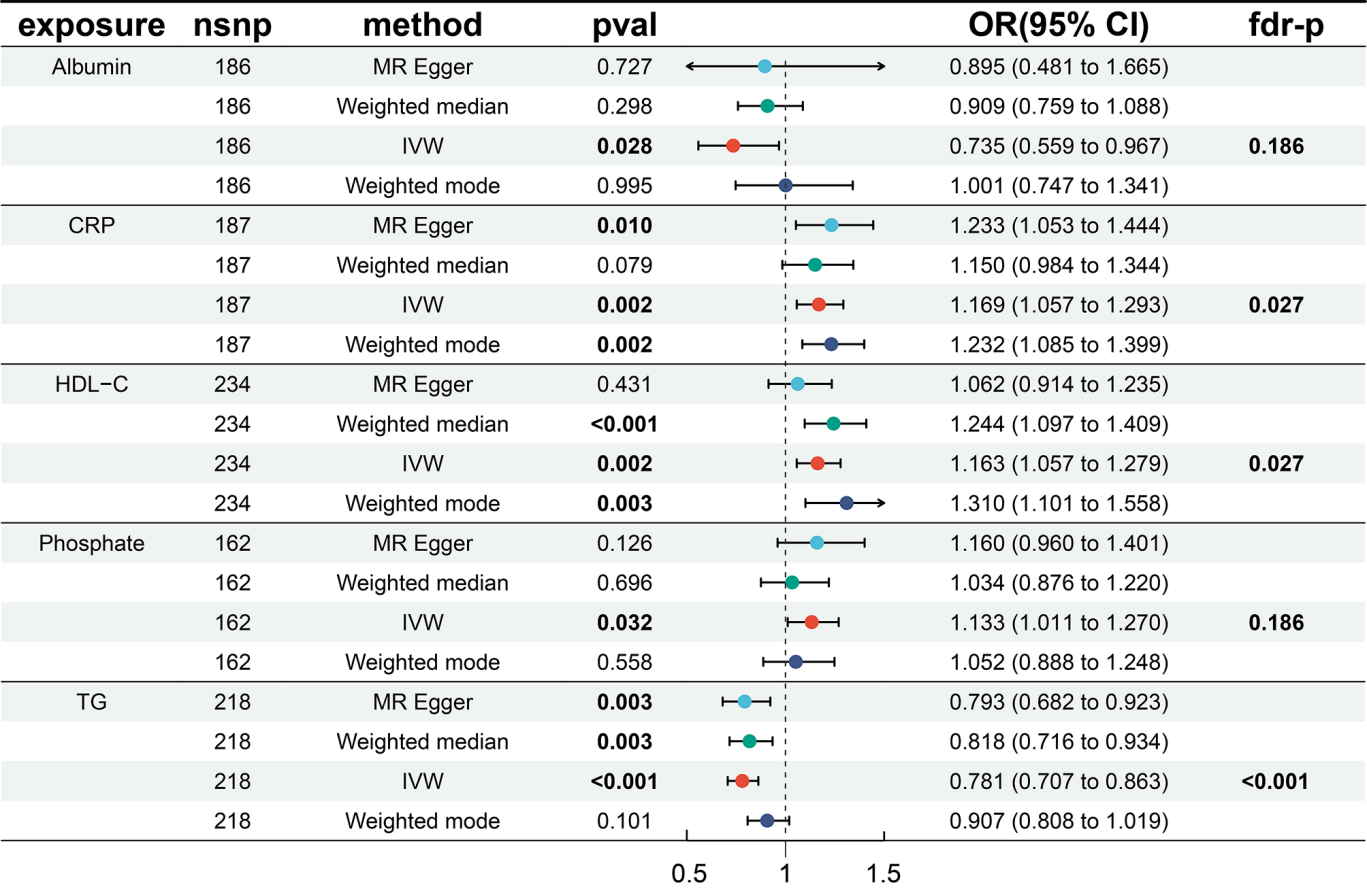
The MR result of biomarkers with significant effect with AMD

We first analyzed the causal relationship between 35 biomarkers and mixed AMD (which includes both dry and wet types) using MR analysis. Of the 35 biomarkers, under the criterion of *P* < 0.05, the IVW method found that 6 biomarkers were significantly associated with Mixed AMD, including TG, HDL-C, CRP, ALB, Phosphate, and Vitamin D (Fig. 2). We conducted a false discovery rate (FDR) correction for our results to get the FDR-P, which we consider the robust *P*-value. With an FDR < 0.05, we still found that 3 biomarkers have a significant causal relationship with AMD, and with FDR < 0.2 are 5. Among them, TG (OR = 0.78, 95% CI 0.71–0.86, *p* = 1.08E-6) showed a significant negative causal relationship with AMD, suggesting a protective role in the progression of AMD, while HDL-C (OR = 1.16, 95% CI 1.06–1.28, *p* = 0.0019) and CRP (OR = 1.17, 95% CI 1.07–1.29, *p* = 0.0023) showed a positive causal relationship, indicating a potential increase in the risk of AMD. Consistent results of TG were found by MR analysis using other MR methods, including weighted median (OR = 0.82, 95% CI 0.72–0.93, *p* = 0.003) and MR Egger (OR = 0.79, 95% CI 0.68–0.92, *p* = 0.003). Similarly, the results for HDL-C were doubly validated in both the Weighted mode (OR = 1.31, 95% CI = 1.10–1.56, *p* = 0.0026) and Weighted median (OR = 1.24, 95% CI = 1.10–1.41, *p* = 0.0006) methods, and the results for CRP were doubly validated in the MR Egger (OR = 1.23, 95% CI = 1.05–1.44, *p* = 0.01) and Weighted mode (OR = 1.23, 95% CI = 1.09–1.40, *p* = 0.0015) methods  (Fig. 3). In addition to these three, IVW also identified that Albumin has a negative causal relationship with AMD, and Phosphate and Vitamin D have positive causal relationships with AMD. However, their significance is greater than 0.05 in the FDR, suggesting that the causal relationships may not be strong. We did not find heterogeneity and horizontal pleiotropy in these results.

### Causal Effect of Biomarkers with AMD subtypes in MR

To further explore the causal relationships between these biomarkers and different types and stages of AMD, we conducted MR studies on dry, wet, and early-stage AMD separately, hoping to gain more support for the use of biomarkers in early diagnosis, typing, and indication of disease progression in AMD (Table 1). TG exhibited a significant causal relationship in both dry and wet forms of AMD, and both were negative, suggesting that TG has a significant protective effect against both dry and wet AMD. Furthermore, LDL-C also exhibited a significant negative causal relationship in dry AMD, similarly suggesting a potential protective effect against dry AMD. On the other hand, CRP showed a negative causal relationship in dry AMD, while Vitamin D exhibited a significant positive causal relationship in wet AMD, suggesting its possible involvement in the progression of wet AMD.

The MR analysis results for early-stage AMD presented a variety of cases. Using the IVW method, 6 biomarkers were found to have significant causal relationships, which are ALB, ApoA1, ApoB, Calcium, HDL-C, TG. After correction with FDR adjusted, ALB (OR = 1.17, 95% CI 1.05∼1.30, *p* = 0.0037), TG (OR = 0.79, 95% CI 0.72∼0.88, *p* < 0.0001), HDL-C (OR = 1.17, 95% CI 1.05∼1.30, *p* = 0.0037) still showed a significant causal relationship with early-stage AMD, leading us to believe the association is relatively strong. We did not find heterogeneity and horizontal pleiotropy in these results.

### Baseline Characteristics of Population-based Study from NHANES

In this investigation, a total of 2,534 adults aged over 40 were selected according to specific inclusion and exclusion criteria. The average age of the participants was 59.64 years, with a standard deviation of 11.34 years. Of the cohort, 50.3% were male and 49.7% were female. The ethnic breakdown was as follows: 55.4% non-Hispanic white, 18.5% non-Hispanic black, 15.5% Mexican American, with the remaining 10.5% comprising other ethnicities. Building on prior MR studies, we assessed the relationship between CRP, TG, and HDL-C, and AMD within this demographic. The average concentrations of TG, CRP, and HDL-C were measured at 1.44 mmol/L, 1.43 mmol/L, and 0.48 mg/dL, respectively. Regarding biomarkers, significant differences were observed in clinical laboratory examinations between the AMD group and the control group for TG, HDL-C, and CRP, while differences in other biomarkers did not reach statistical significance. Specifically, the levels of TG were significantly lower in the AMD group compared to the control group, with the lowest levels observed in the late AMD group. On the other hand, HDL-C and CRP levels were significantly higher in the AMD group and reached their highest in the late group. In the analysis among early AMD, late AMD, and the control group, ALB also showed variability, with levels significantly lower in the late group compared to both the control and early group. The results from the NHANES study were consistent with the findings of the previous MR analysis.

### Association between TG, CRP, HDL-C and AMD

Table 2 displays the outcomes from the multivariate regression analysis. In the unadjusted model, HDL-C [0.31 (0.06∼0.61), *p* = 0.016] was strongly associated with AMD. Yet, this significant positive correlation turned insignificant in Model 2 after adjustments for gender, age, and race variables were made. Following adjustments for all covariates, the relationship between HDL-C and AMD shifted to significant in Model 3 [0.38 (0.05∼0.66), *p* = 0.011]. CRP was significantly associated with AMD across all three models: non-adjusted [1.23 (1.08∼1.40), *p* = 0.012], adjusted by age, gender, and race [1.23 (01.05∼1.42), *p* = 0.005], and adjusted by all covariates [ 1.24 (1.07∼1.44), *p* = 0.005], showing a significant correlation with AMD. Similarly, TG exhibited a significant correlation with AMD in all three models [ -0.17 (-0.40 ∼ -0.07), *p* = 0.023], [− 0.36 (-0.61 ~ -0.08), *p* = 0.011 ], [ -1.02 (-1.46∼− 0.58), *p* < 0.001 ], displaying a negative relationship, suggesting that TG may play a protective role in AMD (Table 3). These findings are consistent with the results from previous MR studies.

**Table 1:** The MR result of biomarkers with AMD subtypes

Biomarker	Phenotype	nSnp	Method	Or(95% CI)	Pval	Fdr-*P*
TG	dAMD	218	Weighted median	0.75 (0.64∼0.88)	**0.0005**	
			IVW	0.76 (0.68∼0.85)	**< 0.0001**	**0.0001**
			Weighted mode	0.85 (0.73∼0.99)	**0.0388**	
CRP	dAMD	187	Weighted median	1.24 (1.05∼1.47)	**0.0111**	
			IVW	1.17 (1.04∼1.31)	**0.0024**	**0.0396**
			Weighted mode	1.27 (1.10∼1.47)	**0.0017**	
LDL-C	dAMD	218	Weighted median	0.90 (0.79∼1.04)	0.1478	
			IVW	0.87 (0.79∼0.96)	**0.0035**	**0.0396**
			Weighted mode	0.90 (0.79∼1.03)	0.1221	
TG	wAMD	210	Weighted median	0.90 (0.76∼1.07)	0.2427	
			IVW	0.79 (0.70∼0.90)	**0.0004**	**0.0149**
			Weighted mode	0.91 (0.78∼1.07)	0.2528	
VitaminD	wAMD	65	Weighted median	1.65 (1.28∼2.11)	**0.0001**	
			IVW	1.41 ( 1.10∼1.81)	**0.0022**	**0.0394**
			Weighted mode	1.50 (1.21∼1.85)	**0.0004**	
ALB	eAMD	106	Weighted median	0.92 (0.75∼1.13)	0.4541	
			IVW	0.84 (0.73∼0.96)	**0.0041**	**0.0463**
			Weighted mode	0.91 (0.66∼1.25)	0.5502	
TG	eAMD	112	Weighted median	0.83 (0.73∼0.94)	**0.0044**	
			IVW	0.79 (0.72∼0.88)	**< 0.0001**	**< 0.0001**
			Weighted mode	0.82 (0.73∼0.92)	**0.0007**	
HDL-C	eAMD	121	Weighted median	1.17 (0.98∼1.39)	0.0745	
			IVW	1.17 (1.05∼1.30)	**0.0037**	**0.0463**
			Weighted mode	1.21 (0.99∼1.47)	0.0632	

**Table 2:** Weighted characteristics of the study population based on AMD

Characteristic	AMD		*P*	Phenotype		*P*
	No AMD	AMD		Early AMD	Late AMD	
N	2325	209		181	28	
Age	58.6 ± 11.9	71.1 ± 11.3	**< 0.001**	70.0 ± 11.5	77.9 ± 5.8	**< 0.001**
SEX(%)			0.442			0.151
Female	1148 (49.3%)	109 (52.2%)		90 (49.7%)	19 (67.9%)	
Male	1177 (50.7%)	100 (47.8%)		91 (50.3%)	9 (32.1%)	
RACE(%)			**< 0.001**	27 (14.9%)	0 (0.0%)	**< 0.001**
Mexican	363 (15.6%)	27 (12.9%)		27 (14.9%)	0 (0.0%)	
Non-Hispanic White	1249 (53.7%)	156 (74.6%)		130 (71.8%)	26 (92.8%)	
Non-Hispanic Black	461 (19.8%)	10 (4.8%)		10 (5.5%)	0 (0.0%)	
Other Hispanic	177 (7.6%)	9 (4.3%)		9 (4.9%)	0 (0.0%)	
Other Race	75 (3.2%)	7 (3.3%)		5 (2.7%)	2 (7.1%)	
BMI	29.2 ± 6.5	28.1 ± 5.5	**0.014**	28.3 ± 5.6	26.3 ± 4.9	**0.013**
EDUCATION			0.106			0.461
HBP(%)			0.086			0.109
Yes	1065 (45.9%)	112 (53.6%)		93 (51.4%)	19 (67.9%)	
No	1260 (54.1%)	97 (46.4%)		88 (48.6%)	9 (32.1%)	
DIABETES(%)			0.551			0.814
Yes	355 (15.3%)	30 (14.4%)		25 (13.8%)	5 (17.9%)	
No	1918 (82.4%)	172 (82.3%)		150 (82.9%)	22 (78.6%)	
Unknow	49 (2.1%)	6 (2.9%)		5 (2.8%)	1 (3.6%)	5 (2.8%)
CHO	5.2 ± 1.0	5.1 ± 1.1	0.541	5.1 ± 1.0	5.5 ± 1.3	0.295
TG	1.4 ± 0.7	1.3 ± 0.6	**0.023**	1.3 ± 0.6	1.2 ± 0.5	**0.045**
LDL-C	3.1 ± 0.9	2.9 ± 0.9	0.058	2.9 ± 0.9	3.1 ± 1.1	0.128
HDL-C	1.4 ± 0.4	1.5 ± 0.4	**0.016**	1.5 ± 0.5	1.7 ± 0.4	**0.003**
CRP	0.5 ± 0.9	0.6 ± 1.3	**0.012**	0.6 ± 1.3	0.8 ± 1.8	**0.021**
HbA1C	5.9 ± 2.1	5.8 ± 1.6	0.702	5.8 ± 1.6	5.9 ± 2.0	0.906
ALB	4.1 ± 0.3	4.1 ± 0.3	0.186	4.2 ± 0.3	4.0 ± 0.4	**0.009**
ApoB	99.8 ± 24.9	96.6 ± 23.6	0.081	96.4 ± 22.7	98.2 ± 28.4	0.204
INSULIN	75.6 ± 73.6	70.6 ± 61.5	0.334	72.4 ± 63.9	58.4 ± 41.9	0.196

Mean ± SD for continuous variables: *P* value was calculated by weighted linear regression model

% for categorical variables: *P* value was calculated by weighted chi-square test

BMI, body mass index; LDL- C, low-Density Lipoprotein Cholesterol; HDL-C, high-Density Lipoprotein Cholesterol;

ALB, albumin; Apo, apolipoprotein;

CRP, c reactive protein; CHO, cholesterol; HbA1C, glycated haemoglobin; TG, triglycerides

**Table 3:** The association between Biomarkers and AMD

Biomarker	Model 1		Model 2		Model 3	
	Beta (95% CI)	P	Beta	P	Beta	P
HDL-C	0.31 (0.06∼0.62)	0.016	0.19 (− 0.09∼0.41)	0.383	0.38 (0.05∼0.66)	0.011
CRP	1.23 (1.08∼1.40)	0.012	1.23 (1.05∼1.42)	0.005	1.24 (1.07∼1.44)	0.005
TG	-0.17 (-0.40 ~ -0.07)	0.023	-0.36 (-0.61 ~ -0.08)	0.011	-1.02 (-1.46 ~ -0.58)	< 0.001

Model 1: Non-adjusted

Model 2: Adjust by age, sex, race

Model 3: Adjust for: age, sex, race, bmi, education, HBP, Diabetes, CHO, LDL-C, HBA1C, ALB, APO, Insulin

**Fig. 4:**
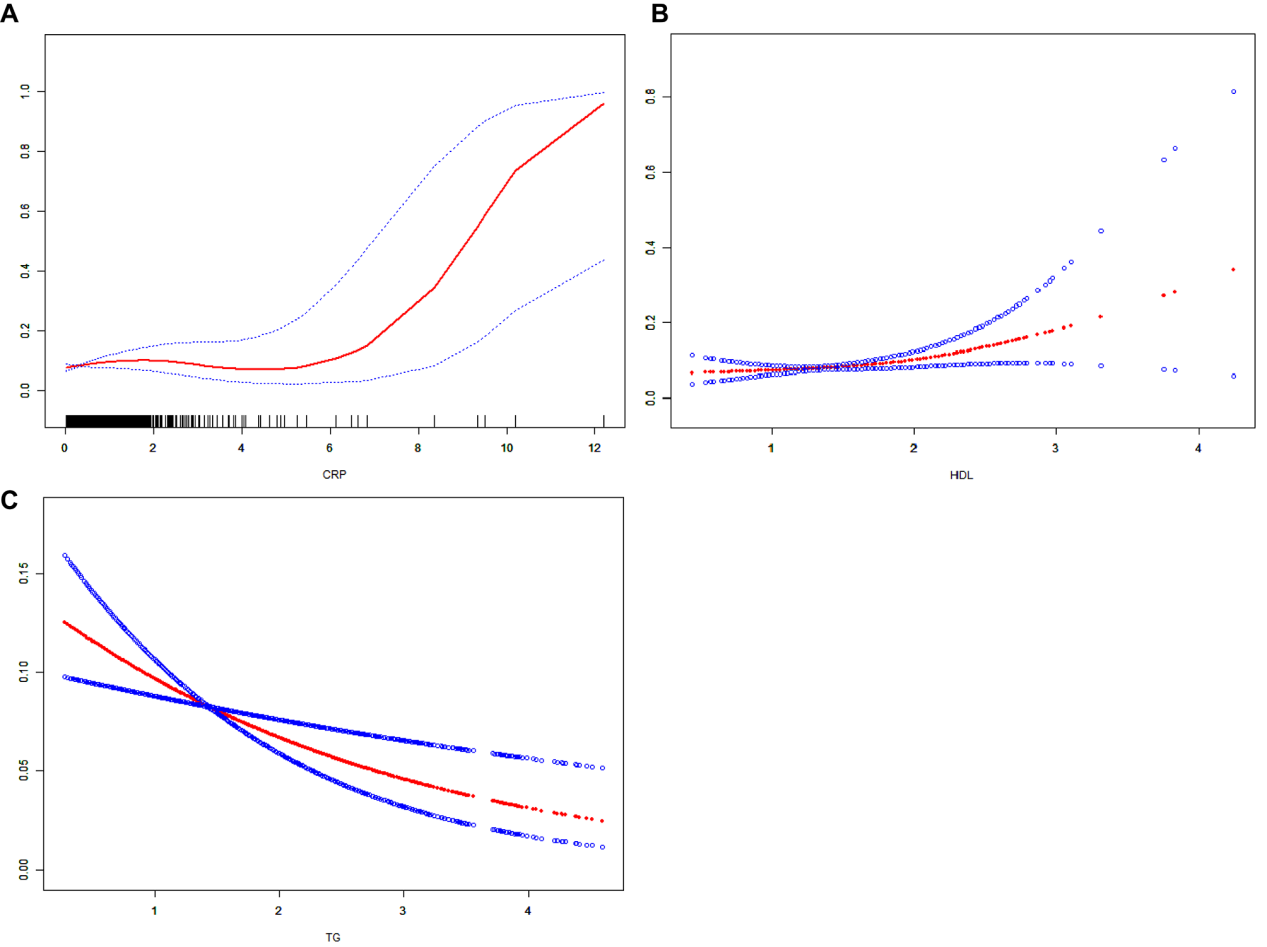
The association between biomarkers and AMD. **A** The red line represents the smooth curve fit between variables. Blue bands represent the 95% confidence interval from the fit. This is the curve of CRP. **B** This is the curve of HDL-C. **C** This is the curve of TG

We performed a smooth curve fit to describe the nonlinear relationship between three biomarkers and AMD  (Fig. 4). Using a two-segment linear regression model, we found a nonlinear relationship between HDL-C, TG and AMD with an inflection point of 1.91 mmol/L and 0.742 mmol/L. Then we found a U-shaped curve was present in CRP and AMD, with inflection points of 6.5 mg/dL.

## Discussion

In this study, we used MR analysis and cross-section study to the association of biomarkers and AMD subtypes. Our findings indicate protective effects for TG and pathogenic effects of HDL-C and CRP. Causal effects for some biomarkers like pathogenic effects of Vitamin D in wet AMD and protective roles of LDL-C in dry AMD were also highlighted. 6 biomarkers were found to have significant causal relationships, which are ALB, ApoA1, ApoB, Calcium, HDL-C, TG. Our study is the first to combine MR analysis related to biomarkers with a cross-sectional study based on the NHANES database, conducting a systematic analysis of the causal relationships and correlations between various biomarkers and AMD. By corroborating findings, the reliability of our study results has been doubly verified, suggesting that TG, CRP, and HDL-C have the potential to become important indicators in the clinical diagnosis of AMD, as well as in the assessment of treatment efficacy and disease progression. In early AMD, lipid metabolism-related biomarkers are more active compared to late AMD, but CRP shows no significant association. This suggests that early AMD may be closely related to lipid metabolism, while late AMD exhibits a stronger inflammatory response.

TG have been reported in previous studies to play a protective role in AMD [24, 25], and both our MR study and cross-sectional analysis have confirmed this. Furthermore, by researching different subgroups of AMD, we found that triglycerides exhibit a significant protective effect in both dry and wet forms of AMD, with an even more pronounced effect in dry AMD. Previous studies have unveiled the presence of lipoproteins within Bruch’s membrane, identifying a notable density of systemic LDL, HDL, and very-low-density lipoproteins [26]. The role of lipids and lipoproteins in forming extracellular lesions, such as basal deposits and drusen (localized accumulations of extracellular debris rich in lipids), in the aging Bruch’s membrane is well-documented [27–29]. Numerous studies have delved into the link between lipid profiles and the propensity for developing AMD [30]. Additionally, several epidemiologic investigations have highlighted a significant correlation between elevated HDL levels and an augmented risk of soft drusen, neovascular AMD, and geographic atrophy [31, 32]. Despite these findings, the direct impact of systemic lipids on AMD, or their reflection of lipid metabolism within the retina, remains to be conclusively determined. In our study utilizing the NHANES cohort, we compared lipid levels (HDL-C, LDL-C, ApoB, CHO, TG) between AMD cases and controls. Statistical adjustments revealed significantly lower TG levels in AMD individuals compared to controls, and elevated HDL-C levels in both early and late stages of AMD, highlighting the nuanced relationship between lipids and AMD risk. Our cross-sectional analysis showed no significant differences in CHO, LDL-C, and ApoB levels between AMD cases and controls. A previous MR studies have revealed that LDL-C, ApoB, and CHOL are associated with a reduced risk of AMD [13]. Our MR analysis also identified differences in CHO, LDL-C, and ApoB for dry AMD, not seen in wet AMD, however, they became non-significant after FDR adjustment. Our cross-sectional study based on the NHANES population also confirmed this. We used smoothing curve fitting to study the possible trends of TG and HDL in AMD, finding that an increase in TG can effectively reduce the risk of AMD, while HDL-C has the opposite effect. Interestingly, the association between CRP and AMD shows a U-shaped curve, suggesting a more complex relationship between CRP and AMD. In the range below 6.5 mg/dL, the association between CRP and AMD is not evident. However, above this threshold, the level of CRP and the risk of developing AMD sharply increase, indicating the role of inflammatory responses in AMD. These findings underscore the unclear linkage between specific lipids and AMD, necessitating further large-scale and animal research to delve into lipid metabolism’s role in AMD, especially considering subtypes [33–35]. Significantly, TG’s protective causal role in AMD suggests its promise as a future therapeutic target, particularly for dry AMD, and HDL-C could also become a therapeutic target for AMD.

Previous studies have found that CRP levels are significantly elevated in patients with AMD, especially in those with wet AMD [36, 37]. A study revealed that CRP as a mediator of complement activation and inflammatory signaling to increase the risk of AMD [14]. In our study, we found that CRP, despite having a significant causal relationship with AMD, particularly dry AMD, and significantly increasing the risk of AMD, does not show a significant causal relationship with early AMD [38]. Previous research has shown that some lipid metabolism-related biomarkers are causally related to early-stage AMD [25]. Our study is the first to comprehensively find that ApoA, ApoB, HDL-C, and TG all have a certain causal relationship with early-stage AMD. Among these, ApoA and HDL-C suggest an increased risk of AMD, while TG and ApoB are associated with a reduced risk of AMD. A study has shown that CHO is related to the risk of developing early-stage AMD, but this was not confirmed in our study [39]. The main clinical feature of early AMD is the appearance of drusen, which is directly related to lipid metabolism [40]. Therefore, combined with our research results, the role of lipid metabolism in the development of early AMD is further confirmed. Inflammatory responses, however, are more pronounced in late AMD.

A previous study has observed an association between blood calcium levels and early AMD [41], our research has confirmed that calcium can effectively reduce the risk of early AMD. Currently, there are no direct studies that reveal how blood calcium regulates AMD, nor has its protective role in AMD been directly proven. However, research has found that osteoporosis is associated with early AMD and can increase the risk of early AMD, but it has no association with late AMD [42]. A study has found that calcium deposits are abundant in sub-retinal pigment epithelial (RPE) deposits, suggesting that calcium may play an important role in the pathogenesis of AMD [43]. More research is needed in the future to investigate the role of calcium in AMD, especially its function in early AMD.

Our study has several advantages. First, we are the first to combine biomarker MR analysis with cross-sectional studies based on the NHANES population to validate the biomarkers identified by MR studies, enhancing the accuracy and credibility of our research. Second, we further confirmed that TG can effectively reduce the risk of AMD, while HDL-C can increase the risk of AMD, and CRP can significantly increase the risk of dry AMD, especially in its later stages. Third, we analyzed the association between biomarkers and different stages of AMD, finding that lipid metabolism-related biomarkers are more closely associated with early-stage AMD, while inflammatory responses have a closer causal relationship and association with late-stage AMD. Fourth, our research model is scalable and can be used in the future to study more complex eye diseases and other systemic diseases.

Our study is also subject to several limitations. Firstly, our analysis was conducted using data from the European population, which may limit its generalizability. Secondly, despite efforts to identify and eliminate outlier variants, the potential for horizontal pleiotropy to affect our findings cannot be entirely ruled out. Thirdly, although we studied 35 biomarkers with different subtypes of AMD, there are still significant limitations with these biomarkers and the GWAS data for AMD. More biomarkers need to be included in future research. Fourth, our study’s findings should be approached with caution due to its cross-sectional nature, which doesn’t establish temporality, and potential unaccounted confounding factors, despite adjustments. Finally, the lack of data on participants’ information in the NHANES database, limiting the number of AMD patients, especially those with late-stage AMD, included in the study has a certain impact on the accuracy of the research results.

## Conclusion

Our findings suggest that higher levels of TG may lower the risk of AMD, while elevated levels of HDL-C are associated with an increased risk of AMD. Additionally, CRP levels are linked with a heightened risk of wet AMD, the lipid-related biomarkers are crucial in early AMD stages, and CRP’s role becomes more prominent in the risk of late AMD. To confirm our findings, more large-scale prospective investigations and basic experiments are needed.

### Electronic Supplementary Material

Below is the link to the electronic supplementary material.

Supplementary Material 1

Supplementary Material 2

Supplementary Material 3

Supplementary Material 4

Supplementary Material 5

Supplementary Material 6

Supplementary Material 7

Supplementary Material 8

Supplementary Material 9

## Data Availability

No datasets were generated or analysed during the current study.
